# Impact of tablet’s correlated color temperature on ocular and visual function in myopic children: a pilot study

**DOI:** 10.3389/fmed.2026.1804663

**Published:** 2026-05-13

**Authors:** Yunyun Chen, Lili Mei, Yanting Feng, Beilei Zhang, Changbiao Xu, Jian Jiang, Jingwei Zheng, Jinhua Bao, Jingjing Xu

**Affiliations:** 1 National Clinical Research Center for Ocular Diseases, Eye Hospital, Wenzhou Medical University, Wenzhou, China; 2 National Engineering Research Center of Ophthalmology and Optometry, Eye Hospital, Wenzhou Medical University, Wenzhou, China

**Keywords:** accommodation, break-up time of tear film, correlated color temperature, near-work induced transient myopia, tablet

## Abstract

**Purpose:**

This study aimed to determine whether a tablet’s correlated color temperature (CCT) setting affects ocular and visual function in myopic children.

**Methods:**

A total of 24 myopic children participated in this study. They were asked to watch a 30-min video at a distance of 33 cm on tablets with low (2800 K) and high CCT (8500 K) settings, in a random order on two different days. Near work-induced transient myopia (NITM), binocular vision function, tear break-up time (TBUT), and asthenopia questionnaire responses were evaluated before and immediately after the 30-min video-watching task.

**Results:**

A significant interaction (CCT setting*examination time) (*F* = 6.01, *p* = 0.02) was observed for NITM, showing a trend toward an increase in the average initial NITM from pre- to post-video viewing when using the high-CCT tablet compared to the low-CCT tablet. No significant interaction was observed for the other visual function, TBUT, and asthenopia symptom scores (all *p* > 0.05), although the 30-min task using the tablet did result in a reduction in TBUT and an exacerbation of some asthenopia symptoms (*p* < 0.05).

**Conclusion:**

The 30-min tablet usage task decreased tear film stability and exacerbated some asthenopia symptoms but did not significantly affect other visual functions, regardless of the screen’s CCT setting. The use of a high-CCT (8500 K) tablet induced a slightly, but significantly, higher initial NITM compared to a low-CCT (2800 K) tablet; however, given the small magnitude of this difference, its clinical relevance still needs to be confirmed.

## 1 Introduction

Myopia has become a significant public health concern worldwide, particularly in China. Sustained near work (1) and decreased outdoor activity (2) are considered two crucial environmental factors in the onset and development of myopia. Increased time spent in education is also a causal risk factor for myopia (3). With the development of the social economy and the transformation of educational approaches, the usage time of screen-based equipment (SBE) among children for studying has been increasing steadily. Particularly during the COVID-19 pandemic, more than 220 million children participated in online learning from home. With the increased use of SBE (4) and reduced outdoor activity, the incidence of myopia among children has increased rapidly (5).

In addition to the adverse impact on juvenile myopia onset and progression, the use of SBE (e.g., cell phones and computers) also affects ocular health. SBE use could reduce the blink rate, increase incomplete blinks, and lower tear meniscus height, leading to tear film instability with a reduced tear break-up time (TBUT), ultimately resulting in dry eye (6–8). Moreover, the accommodative and vergence system remains continuously engaged during the prolonged use of SBE. Data from several studies suggest that the use of SBE could decrease binocular accommodative facility (9), reduce accommodative amplitude, and cause a distal shift in the near point of convergence (NPC) (10, 11). The participants in studies examining the effects of SBE on visual function are predominantly adults (12), rather than children. Moreover, near work can induce a small amplitude, transient myopic shift in the refractive state, known as near work-induced transient myopia (NITM) (13, 14), which is one of the key parameters used to characterize the accommodative response following sustained near work tasks. Nevertheless, whether the utilization of SBE exerts any effect on NITM remains unknown. In addition to causing dry eyes and changes in visual function, SBE can also lead to subjective symptoms of asthenopia, which are associated with the duration of SBE use (15, 16).

Therefore, parents of myopic children often express concern that the use of SBE may further impair their children’s vision, and manufacturers also seek to determine whether variations in screen settings differentially affect pediatric visual health. Correlated color temperature (CCT) is a critical index of display performance. Low CCT has a higher proportion of long-wavelength red light, whereas high CCT has a higher proportion of short-wavelength blue light. The impact of low-intensity, short-wavelength visible light emitted by digital devices on the eye has been a subject of controversy (17–19). A recent study reported that long-term exposure to low-illuminance blue light from phones may cause structural and functional damage to retinal tissue (20). Moreover, short-wavelength visible light also affects the ocular surface (21, 22) by inducing epithelial cell death and altering the expression of inflammatory genes, thereby contributing to dry eye or exacerbating symptoms of visual fatigue (23). Consequently, reducing exposure to low-intensity, short-wavelength visible light is considered beneficial for ocular health. As a result, device settings that reduce the proportion of short-wavelength visible light—such as night mode—are becoming increasingly popular. Night mode is primarily achieved by lowering the screen’s CCT. Nevertheless, the effect of a screen’s CCT setting on ocular and visual function still needs to be determined. To address this gap, we conducted a randomized controlled trial to investigate the impact of the tablet’s CCT on ocular and visual function in children.

## 2 Materials and methods

### 2.1 Participants

A total of 24 myopic children were recruited to participate. The following criteria were applied: Age 8–12 years; spherical equivalent between −0.75 and −4.00 D; astigmatism <0.50 D in either eye; anisometropia <1.00 D; monocular best-corrected visual acuity >0.00 LogMAR; distant lateral phoria within −5Δ to +3Δ; near lateral phoria within 0 to −15Δ; near point of accommodation (NPA) greater than (15–0.25*age) D; NPC < 7 cm; and no strabismus, no symptoms of visual fatigue, and no history of any ocular disease or myopia control interventions such as orthokeratology or atropine eye drops.

This study adhered to the tenets of the Declaration of Helsinki and was approved by the Ethics Committee of the Eye Hospital affiliated to Wenzhou Medical University (IRB approval: 2020-143-K-128-01). Informed consent was obtained from all participants and their guardians.

### 2.2 Procedures

**Figure 1:**
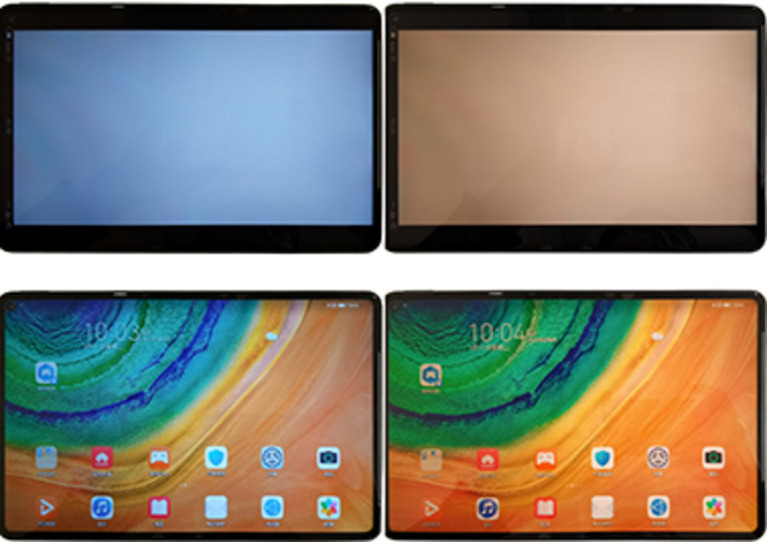
Photos of a whiteboard (top) and a colorful desktop (bottom) displayed on the tablets with high (left) and low (right) CCT settings. CCT: correlated color temperature.

**Figure 2:**
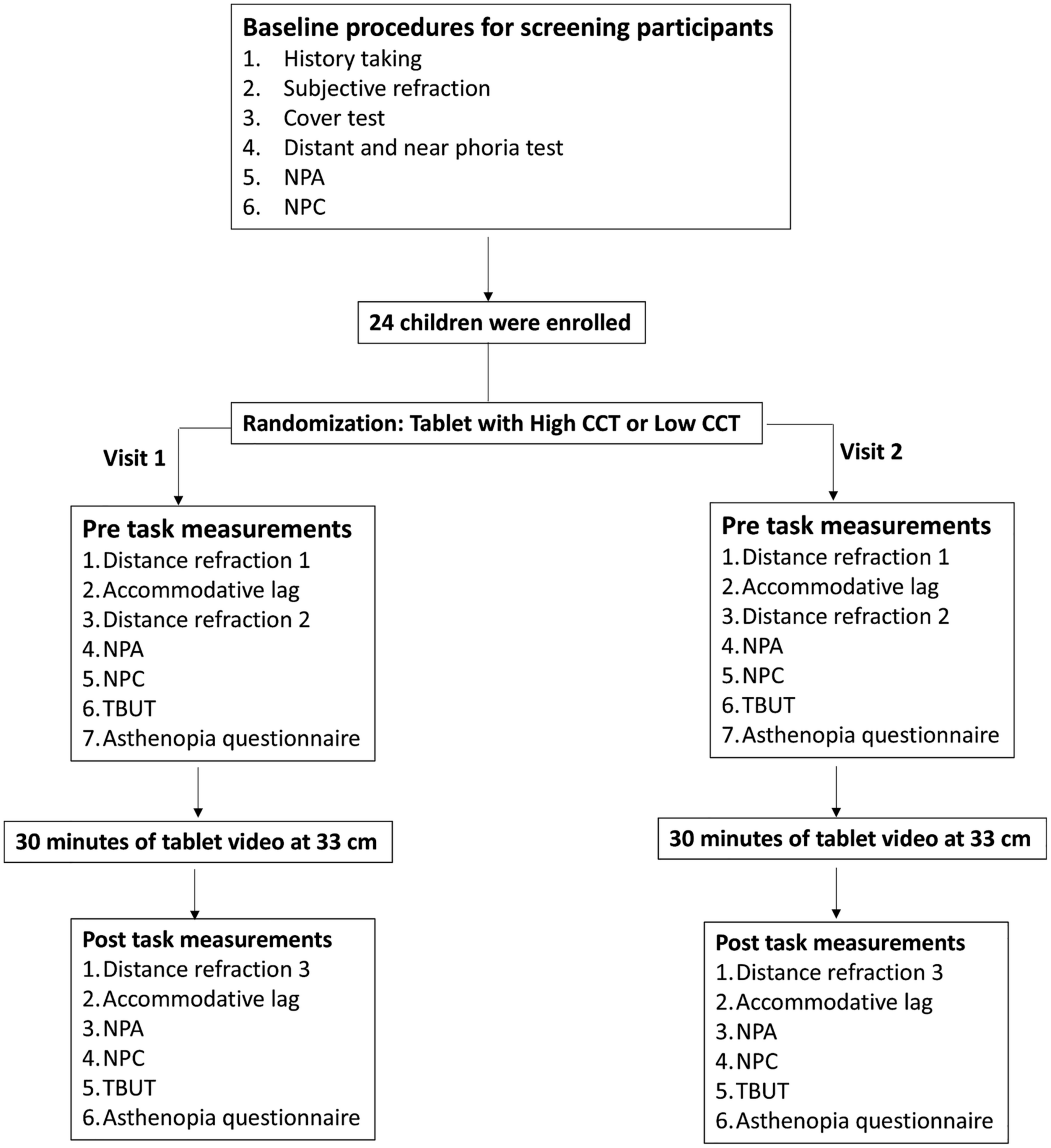
Flowchart of the study protocol. NPA: near point of accommodation, NPC: near point of convergence, CCT: correlated color temperature, TBUT: tear break-up time.

Before the experiments, the CCT of the lighting and the illuminance of the test room were set at 5000 ± 100 K and 300 ± 50 lx, respectively. The CCT of two identical tablets (10.4-inch, Huawei) with a luminance of 160 cd/m^2^ was set to low (2800 K) and high (8500 K), respectively (Figure 1). The chromaticity of the tablet displays under the two CCT settings was measured using a colorimeter (CA-310, Konica Minolta, Japan) with a homogeneous white screen. The measured CIE 1931 xy chromaticity coordinates were (x:0.2892 ± 0.01, y:0.3041 ± 0.01) for the 8500 K setting and (x:0.4519 ± 0.01, y:0.4086 ± 0.01) for the 2800 K setting. The study flowchart is shown in Figure 2. Due to the clear differences in screen settings, masking was not possible for either examiners or participants. The two tasks (low or high CCT) were randomized using a random number table and performed on two different days, with an interval of at least 1 day and no more than 1 week. After the screening procedures, eligible participants underwent a series of pre-task objective measurements within 5 min, including accommodative lag, NITM, NPA, NPC, and TBUT, followed by a subjective asthenopia questionnaire. After the pre-task measurements, the participants were asked to watch a cartoon video of Peppa Pig for 30 min on a tablet placed at 33 cm with a full-corrected trial frame. A chin rest was used to maintain viewing distance and gaze angle during the video task. All measurements were repeated immediately after the video-watching task was completed.

Accommodative lag was measured using an infrared open-field autorefractor (WAM-5500; Grand Seiko, Japan) in high-speed mode at a sampling speed of 5 Hz. With the best distance-corrected trial frame, the participants were instructed to binocularly look at a Maltese target (the visual angle was 10°, and the contrast was 90%) on a tablet at a distance of 33 cm. The autorefractor collected right-eye data for 30 s, and the average value was calculated. Then, the accommodative response and accommodative stimulus were converted to the corneal plane using the effectivity formula (24) to adjust the effect of the spectacle lens on the autorefractor readings.

NITM was also evaluated using the Grand Seiko autorefractor in high-speed mode at a sampling speed of 5 Hz, with participants wearing their best distance-corrected trial frame. Before the measurement, a 3-min fixation at a distance was set to avoid the effect of any previous accommodative after-effects. Then, the autorefractor collected data from the right eye for 3 s while participants looked at the Maltese target at 5.0 m (the visual angle was 2°, and the contrast was 90%). The average was calculated as Distance refraction 1. After accommodative lag was measured at 33 cm, participants were immediately instructed to binocularly look at the Maltese target at 5.0 m, and refractive data were collected again for 10 s. The average was calculated as Distance refraction 2. After 30 min of video watching, Distance refraction 3 was obtained using the same method. The initial NITM magnitude before and after the task was defined as the difference between Distance refraction 2 and 1 and between Distance refraction 3 and 1, respectively.

The NPC and NPA were measured according to the push-up method using the Royal Air Force near point ruler (RAF ruler). The TBUT of the right eye was evaluated with fluorescein under the same illuminance in a slit lamp. Participants were asked to keep their eye open without blinking for as long as possible, and TBUT was recorded with a stopwatch. All measurements were taken twice, and the average value was recorded.

A total of nine symptoms from an asthenopia questionnaire, adapted from the Digital Eye Strain questionnaire (16), were rated on a scale from 0 (none) to 10 (severe). The examiner instructed each participant to complete the questionnaire before and after the task.

### 2.3 Statistical analysis

Data were analyzed using SAS 9.4 statistical software (SAS Institute, Cary, NC, USA). A two-way repeated measures ANOVA was performed to assess between-group differences in low- and high-CCT tablet use, within-group differences between pre- and post-tablet task measurements, and potential interactions. The significance level was set at 5%. *Post hoc t*-tests with Bonferroni correction were performed for pairwise comparisons.

## 3 Results

A total of 24 participants (14 male participants and 10 female participants) were enrolled after screening examinations. Table 1 presents the baseline clinical characteristics. All participants completed the study. Table 2 shows vision function and tear film stability before and after the tablet task.

**Table 1:** The participant’s clinical characteristics.

Demographic Factor	Mean value	Minimum	Maximum
Age (years)	10.4 (1.0)	8	11.8
SER of the right eye (D)	−2.13 (1.00)	−0.75	−4
SER of the left eye (D)	−2.15 (1.04)	−0.75	−4
DLP (Δ)	−1.6 (2.1)	−5	2.5
NLP (Δ)	−5.6 (4.5)	−13.5	0.75
NPA (D)	14.3 (2.1)	11	20
NPC (cm)	5.1 (0.9)	4.5	7

Data are presented as means (SD). SER: spherical equivalent refraction, DLP: distant lateral phoria (exophoria: negative value, esophoria: positive value), NLP: near lateral phoria, NPA: near point of accommodation, NPC: near point of convergence.

**Table 2:** Visual function and tear film stability before and after the tablet task.

Parameters	Low CCT (before)	Low CCT (after)	High CCT (before)	High CCT (after)	CCT setting		Examination time		Interaction (CCT setting*Examination time)	
					*F*-value	*p*-value	F-value	*p*-value	*F*-value	*p*-value
Accommodative lag (D)	1.16 (0.44)	1.06 (0.39)	1.14 (0.42)	1.04 (0.47)	0.38	0.85	1.45	0.24	0.01	0.93
NITM (D)	−0.09 (0.41)	0.01 (0.33)	−0.04 (0.37)	−0.16 (0.44)	1.15	0.30	0.07	0.80	6.01	0.02*
NPA (D)	14.4 (3.5)	14.4 (3.8)	15.4 (3.8)	15.5 (3.5)	6.99	0.01*	0.02	0.88	0.01	0.91
NPC (cm)	6.2 (1.8)	6.6 (2.4)	6.2 (1.6)	6.1 (1.6)	1.30	0.27	2.84	0.11	2.70	0.11
TBUT (s)	7.3 (5.0)	5.2 (3.0)	6.9 (4.2)	5.9 (3.2)	0.06	0.81	6.51	0.02*	2.61	0.12

Data are presented as means (SD). CCT: correlated color temperature, NITM: near work-induced transient myopia, NPA: near point of accommodation, NPC: near point of convergence, TBUT: the tear break-up time.

### 3.1 Initial NITM magnitude

No significant difference in the initial NITM magnitude was observed between the low- and high-CCT tablet use groups (estimated difference: -0.05 D; 95% confidence interval [CI], −0.16 to 0.05; *p* = 0.30), and no difference was observed before and after the 30-min video-viewing task (estimated difference: −0.01 D; 95% CI, −0.13 to 0.10; *p* = 0.80). However, interaction (CCT setting*examination time) exhibited a statistically significant difference (*F* = 6.01, *p* = 0.02). The initial NITM magnitude increased after viewing the 30-min video on a high-CCT tablet relative to a low-CCT tablet (Table 2). However, the induced NITM magnitudes in the present study were quite small (high-CCT tablet: −0.16 ± 0.44 D; low-CCT tablet: 0.01 ± 0.33 D).

### 3.2 Accommodative lag

No significant differences in accommodative lag were observed between the low- and high-CCT tablet use groups (estimated difference: −0.02 D; 95% CI, −0.21 to 0.18; *p* = 0.85). Moreover, no significant difference in accommodative lag was found before and after the 30-min video-viewing task (estimated difference: −0.10 D; 95% CI, −0.27 to 0.07; *p* = 0.24), and no interaction (CCT setting*examination time) (*F* = 0.01, *p* = 0.93) was found. Therefore, 30-min video watching and tablet CCT settings had no statistically significant effect on accommodative lag.

### 3.3 NPA and NPC

No significant differences in the main effect of examination time (before vs. after the 30-min video-watching task) and interaction (CCT setting* examination time) were observed for the NPA (F_1_ = 0.02, p_1_ = 0.88; F_2_ = 0.01, p_2_ = 0.91) and NPC (F_1_ = 0.284, p_1_ = 0.11; F_2_ = 0.70, p_2_ = 0.11), respectively. In other words, the 30-min video-watching task and the CCT setting of tablets did not affect the NPA and NPC significantly.

### 3.4 TBUT

TBUT significantly decreased after the use of the tablet (estimated difference: −1.59 s; 95% CI, −2.88 to −0.30; *p* = 0.02). However, no significant interaction between CCT setting and examination time (*F* = 2.61; *p* = 0.12) or main effect of CCT setting (estimated difference: 0.13 s; 95% CI, −0.94 to 1.20; *p* = 0.81) was observed (Table 2), suggesting that CCT did not affect TBUT significantly..

### 3.5 Asthenopia questionnaire (shown in the Appendix)

Table 3 presents the scores for nine symptoms in the asthenopia questionnaire. No significant between-group (high vs. low CCT setting), within-group (before vs. after the 30-min video-watching task), or interaction (CCT setting*examination time) differences were observed, except that the symptoms listed as items 1, 2, and 6 were more severe after tablet viewing (*p* < 0.05).

**Table 3:** Questionnaire scores before and after tablet use with low- and high-CCT settings.

Item	Low CCT (Before)	Low CCT (After)	High CCT (Before)	High CCT (After)	CCT setting		Examination time		Interaction (CCT setting*Examination time)	
					*F*	*p*	*F*	*p*	*F*	*p*
1. Eye tired	1.2 (1.9)	2.2 (2.5)	1.1 (2.0)	2.2 (2.6)	0.04	0.85	4.93	0.04*	0.01	0.93
2. Eye pain	0.2 (0.5)	1.0 (1.4)	0.5 (0.9)	1.1 (1.7)	1.87	0.19	7.17	0.01*	1.09	0.31
3. Irritation	0.8 (1.5)	0.9 (1.3)	0.8 (1.19)	1.0 (1.6)	0.00	1.00	0.52	0.48	0.26	0.62
4. Tearing	1.4 (2.5)	1.6 (2.5)	1.0 (2.3)	1.0 (2.1)	2.66	0.12	0.40	0.53	0.30	0.59
5. Dry eye	1.0 (2.1)	1.1 (1.9)	1.1 (2.1)	1.3 (1.9)	0.97	0.34	0.35	0.56	0.06	0.81
6. Burning sensation	0.3 (1.2)	0.3 (0.7)	0.3 (1.2)	0.8 (1.7)	1.71	0.20	4.15	0.05*	2.85	0.11
7. Blurred vision	1.3 (1.9)	1.0 (2.0)	0.8 (2.1)	1.0 (1.6)	0.54	0.47	0.01	0.94	0.69	0.41
8. Difficulty in focusing	0.5 (0.9)	0.7 (1.5)	1.0 (1.4)	1.3 (2.4)	3.81	0.06	0.84	0.37	0.06	0.81
9. Eye strain	3.3 (3.5)	2.8 (3.2)	1.9 (2.8)	2.0 (2.5)	3.23	0.09	1.07	0.31	1.80	0.19

Data are presented as means (SD). CCT: correlated color temperature. *p < 0.05.

## 4 Discussion

The purpose of this study was to evaluate visual function, tear stability, and subjective asthenopia questionnaire scores before and after the usage of a tablet with high and low CCT settings in myopic children. The finding indicated that 30-min tablet use shortened TBUT and worsened some asthenopia symptoms but did not induce substantial changes in visual function. The most notable finding from the analysis was that tablet usage with higher CCT tended to induce a higher initial NITM magnitude, suggesting that the impact of CCT should be considered when adjusting screen settings.

NITM refers to a small amplitude, transient myopic shift in the refractive state after near work. In this study, the initial NITM value was lower than that reported by Vasudevan et al. (14) (0.14–0.29 D). This inconsistency could be attributable to differences in methodologies. In the present study, myopic children watched videos at a distance of 33 cm for 30 min, whereas in Vasudevan et al.’s study, adults read books at a distance of 35–40 cm for 2 h. NITM is likely to be more pronounced with longer durations of near work because of the time accumulation effect (14). Another explanation is that watching videos may induce less precise accommodative effort than reading printed, high-contrast text. NITM-induced temporary retinal defocus has been considered a possible myogenic factor (25). Several effective strategies for myopia control have been shown to reduce NITM, including topical 0.01% atropine (26) and dual-focus soft contact lenses (MiSight) (27). Based on evidence from previous studies, this research found that a prolonged period was required for the accommodative system to revert to a normal level after performing a sustained near task on a high-CCT tablet, which may be unfavorable for myopia development. It is difficult to explain this result, but it might be related to the complicated mechanisms underlying the biological effects of combined task-ambient lighting (28). Higher task CCT (8200 K) combined with relatively lower ambient lighting (5000 K) in this study may have led to reduced sympathetic innervation, which has been proposed to increase NITM (25). Another possible explanation for this is that a high-CCT tablet may improve children’s attention (29) and task performance (30), leading to prolonged tension in the ciliary muscle and resulting in higher NITM.

In the present study, no evidence was found to suggest that the 30-min tablet use task influenced accommodative lag. These results are in agreement with those of Collier and Rosenfield (31), who demonstrated no significant changes in accommodative lag, as measured with a Grand Seiko autorefractor, during a 30-min computer task. Moreover, this study found that accommodative lag was unaffected by the task CCT setting. This finding is in agreement with a previous study, which demonstrated that a blue-blocking screen filter had no effect on accommodation. It has been suggested that SBE usage leads to reduced subjective NPA and a receding NPC due to fatigue of accommodation and convergence (12). This differs from the findings presented here, possibly because of the robust accommodation and convergence of children with normal binocular vision during a 30-min near work task.

Most studies (12, 32, 33) have shown that exposure to SBE for more than 1 h could shorten TBUT, especially in SBE workers. Similarly, TBUT was reduced after 30-min tablet use in the present study. Moreover, the eyes appeared to be slightly more fatigued after 30-min tablet use, with significant worsening of three asthenopia symptoms. The findings reported here suggest that the duration of SBE use in children should not exceed 30 min. Moreover, the CCT setting of the tablets did not affect tear film stability or subjective symptoms. These results corroborate the findings (34, 35) of previous studies, which demonstrated that the blue-blocking filter was not effective in reducing symptoms of digital eye strain. Thus, tear film stability and SBE-related asthenopia were likely influenced by tablet use itself rather than by the CCT setting.

This study has some limitations. First, we adopted a relatively short task duration (30 min). It may be easier to induce ocular changes with longer periods of tablet use. However, it has been suggested that children should not use SBE for more than 60 min per day (36). Second, participants without asthenopia symptoms were enrolled in this study, which limits the generalizability of the findings to individuals with visual symptoms. Third, due to the inability to implement masking, the results of this study cannot completely rule out potential bias introduced by the methodology. Fourth, the initial NITM magnitude and its decay (25) were the two key parameters used to describe accommodation after sustained near work. However, NITM decay was not assessed due to time constraints, which may have limited the ability to fully evaluate the effect of the near task on ocular parameters in the present study. Fifth, although we precisely characterized the chromaticity of the display using a colorimeter, the full spectral power distribution (SPD) was not measured. SPD data would have allowed for a more detailed mechanistic interpretation, particularly regarding wavelength-dependent effects on ocular physiology. Future studies should employ spectroradiometric measurements to replicate our findings and provide complete spectral characterization of the display settings. Finally, the sample size was relatively small for exploring the influence of the CCT setting of the tablet on the eye. Further studies with a larger and more diverse cohort, based on this pilot study, should therefore be conducted to provide more robust evidence regarding the impact of CCT on ocular and visual function.

## 5 Conclusion

The use of a high-CCT (8500 K) tablet in myopic children resulted in a slight increase in NITM magnitude, whereas no significant changes were found in other ocular health indicators, leaving its clinical relevance yet to be confirmed. Notably, in myopic children, 30-min tablet use decreased tear film stability and exacerbated asthenopia symptoms, highlighting viewing duration as a potentially more influential factor than CCT.

## Data Availability

The raw data supporting the conclusions of this article will be made available by the authors, without undue reservation.

## Appendix

Asthenopia questionnaire.

Please rate your feeling on a scale from 1 to 10, using whole numbers.

1. Do you feel tiredness in your eyes?
2. Do you feel pain in your eyes?
3. Do you feel eye irritation?
4. Do you feel tearing in your eyes?
5. Do you feel dryness in your eyes?
6. Do you feel a burning sensation in your eyes?
7. Do you feel blurred vision?
8. Do you have difficulty focusing?
9. Do you feel eye strain?

## References

[1] Dutheil F Oueslati T Delamarre L Castanon J Maurin C Chiambaretta F . Myopia and near work: a systematic review and meta-analysis. Int J Environ Res Public Health. (2023) 20:875. doi: 10.3390/ijerph20010875, 36613196 PMC9820324

[2] Medina A. The cause of myopia development and progression: theory, evidence, and treatment. Surv Ophthalmol. (2022) 67:488–509. doi: 10.1016/j.survophthal.2021.06.005, 34181975

[3] Morgan IG French AN Ashby RS Guo X Ding X He M . The epidemics of myopia: aetiology and prevention. Prog Retin Eye Res. (2018) 62:134–49. doi: 10.1016/j.preteyeres.2017.09.004 28951126

[4] Enthoven CA Tideman JWL Polling JR Yang-Huang J Raat H Klaver CCW. The impact of computer use on myopia development in childhood: the generation R study. Prev Med. (2020) 132:105988. doi: 10.1016/j.ypmed.2020.105988, 31954142

[5] Ma M Xiong S Zhao S Zheng Z Sun T Li C. COVID-19 home quarantine accelerated the progression of myopia in children aged 7 to 12 years in China. Invest Ophthalmol Vis Sci. (2021) 62:37. doi: 10.1167/iovs.62.10.37, 34463719 PMC8411864

[6] Cardona G García C Serés C Vilaseca M Gispets J. Blink rate, blink amplitude, and tear film integrity during dynamic visual display terminal tasks. Curr Eye Res. (2011) 36:190–7. doi: 10.3109/02713683.2010.544442, 21275516

[7] Kamøy B Magno M Nøland ST Moe MC Petrovski G Vehof J . Video display terminal use and dry eye: preventive measures and future perspectives. Acta Ophthalmol. (2022) 100:723–39. doi: 10.1111/aos.15105, 35122403 PMC9790652

[8] Cartes C Segovia C Salinas-Toro D Goya C Alonso MJ Lopez-Solis R . Dry eye and visual display terminal-related symptoms among university students during the coronavirus disease pandemic. Ophthalmic Epidemiol. (2022) 29:245–51. doi: 10.1080/09286586.2021.1943457, 34251964

[9] Golebiowski B Long J Harrison K Lee A Chidi-Egboka N Asper L. Smartphone use and effects on tear film, blinking and binocular vision. Curr Eye Res. (2020) 45:428–34. doi: 10.1080/02713683.2019.1663542, 31573824

[10] Kurimoto S Iwasaki T Nomura T Noro K Yamamoto S. Influence of VDT (visual display terminals) work on eye accommodation. J UOEH. (1983) 5:101–10. doi: 10.7888/juoeh.5.101 6679614

[11] Lan M Yang XB Liu LQ. Comparative study on visual fatigue caused by watching liquid crystal display and projection display. Zhonghua Yan Ke Za Zhi. (2019) 55:595–600. doi: 10.3760/cma.j.issn.0412-4081.2019.08.009, 31422638

[12] Jaiswal S Asper L Long J Lee A Harrison K Golebiowski B. Ocular and visual discomfort associated with smartphones, tablets and computers: what we do and do not know. Clin Exp Optom. (2019) 102:463–77. doi: 10.1111/cxo.12851, 30663136

[13] Ciuffreda KJ Vasudevan B. Effect of nearwork-induced transient myopia on distance retinal defocus patterns. Optometry. (2010) 81:153–6. doi: 10.1016/j.optm.2009.03.022, 20211445

[14] Vasudevan B Ciuffreda KJ. Additivity of near work-induced transient myopia and its decay characteristics in different refractive groups. Invest Ophthalmol Vis Sci. (2008) 49:836–41. doi: 10.1167/iovs.07-0197, 18235035

[15] Larese Filon F Drusian A Ronchese F Negro C. Video display operator complaints: a 10-year follow-up of visual fatigue and refractive disorders. Int J Environ Res Public Health. (2019) 16:2501. doi: 10.3390/ijerph16142501, 31337021 PMC6678724

[16] Alabdulkader B. Effect of digital device use during COVID-19 on digital eye strain. Clin Exp Optom. (2021) 104:698–704. doi: 10.1080/08164622.2021.1878843, 33689614

[17] Cougnard-Gregoire A Merle BMJ Aslam T Seddon JM Aknin I Klaver CCW . Blue light exposure: ocular hazards and prevention-a narrative review. Ophthalmol Ther. (2023) 12:755–88. doi: 10.1007/s40123-023-00675-3, 36808601 PMC9938358

[18] Wolffsohn JS Lingham G Downie LE Huntjens B Inomata T Jivraj S . TFOS lifestyle: impact of the digital environment on the ocular surface. Ocul Surf. (2023) 28:213–52. doi: 10.1016/j.jtos.2023.04.004, 37062428

[19] Dain SJ. The blue light dose from white light emitting diodes (LEDs) and other white light sources. Ophthalmic Physiol Opt. (2020) 40:692–9. doi: 10.1111/opo.12713, 32691888

[20] Li H Zhang M Wang D Dong G Chen Z Li S . Blue light from cell phones can cause chronic retinal light injury: the evidence from a clinical observational study and a SD rat model. Biomed Res Int. (2021) 2021:3236892. doi: 10.1155/2021/3236892, 34055970 PMC8147535

[21] Marek V Mélik-Parsadaniantz S Villette T Montoya F Baudouin C Brignole-Baudouin F . Blue light phototoxicity toward human corneal and conjunctival epithelial cells in basal and hyperosmolar conditions. Free Radic Biol Med. (2018) 126:27–40. doi: 10.1016/j.freeradbiomed.2018.07.012, 30040995

[22] Lee HS Cui L Li Y Choi JS Choi JH Li Z . Influence of light emitting diode-derived blue light overexposure on mouse ocular surface. PLoS One. (2016) 11:e0161041. doi: 10.1371/journal.pone.0161041, 27517861 PMC4982597

[23] Rosenfield M. Computer vision syndrome: a review of ocular causes and potential treatments. Ophthalmic Physiol Opt. (2011) 31:502–15. doi: 10.1111/j.1475-1313.2011.00834.x, 21480937

[24] Mutti DO Jones LA Moeschberger ML Zadnik K. AC/a ratio, age, and refractive error in children. Invest Ophthalmol Vis Sci. (2000) 41:2469–78. doi: 10.1016/S0002-9394(00)00760-1, 10937556

[25] Ciuffreda KJ Vasudevan B. Nearwork‐induced transient myopia (NITM) and permanent myopia – is there a link? Ophthalmic Physiol Opt. (2008) 28:103–14. doi: 10.1111/j.1475-1313.2008.00550.x, 18339041

[26] Guo L Fan L Tao J Hua R Yang Q Gu H . Use of topical 0.01% atropine for controlling near work-induced transient myopia: a randomized, double-masked, placebo-controlled study. J Ocul Pharmacol Ther. (2020) 36:97–101. doi: 10.1089/jop.2019.0062, 31800355

[27] Jiménez R Redondo B. Impact of dual-focus soft contact lens wear on near work-induced transient myopia. Clin Exp Optom. (2023) 106:296–302. doi: 10.1080/08164622.2022.2029684 35073496

[28] Hayano J Ueda N Kisohara M Yoshida Y Yuda E. Ambient-task combined lighting to regulate autonomic and psychomotor arousal levels without compromising subjective comfort to lighting. J Physiol Anthropol. (2021) 40:8. doi: 10.1186/s40101-021-00258-w, 34372917 PMC8353805

[29] Barkmann C Wessolowski N Schulte-Markwort M. Applicability and efficacy of variable light in schools. Physiol Behav. (2012) 105:621–7. doi: 10.1016/j.physbeh.2011.09.020 22001491

[30] Hartstein LE LeBourgeois MK Berthier NE. Light correlated color temperature and task switching performance in preschool-age children: preliminary insights. PLoS One. (2018) 13:e0202973. doi: 10.1371/journal.pone.0202973, 30161180 PMC6117001

[31] Collier JD Rosenfield M. Accommodation and convergence during sustained computer work. Optometry. (2011) 82:434–40. doi: 10.1016/j.optm.2010.10.013, 21514899

[32] Yazici A Sari ES Sahin G Kilic A Cakmak H Ayar O . Change in tear film characteristics in visual display terminal users. Eur J Ophthalmol. (2015) 25:85–9. doi: 10.5301/ejo.5000525, 25363850

[33] Wu H Wang Y Dong N Yang F Lin Z Shang X . Meibomian gland dysfunction determines the severity of the dry eye conditions in visual display terminal workers. PLoS One. (2014) 9:e105575. doi: 10.1371/journal.pone.0105575, 25144638 PMC4140788

[34] Palavets T Rosenfield M. Blue-blocking filters and digital eyestrain. Optom Vis Sci. (2019) 96:48–54. doi: 10.1097/OPX.0000000000001318, 30570598

[35] Vera J Redondo B Ortega-Sanchez A Molina-Molina A Molina R Rosenfield M . Blue-blocking filters do not alleviate signs and symptoms of digital eye strain. Clin Exp Optom. (2023) 106:85–90. doi: 10.1080/08164622.2021.2018914 35057697

[36] Misawa T Shigeta S Nojima S. Effects of video games on visual function in children. Nihon Eiseigaku Zasshi. (1991) 45:1029–34. doi: 10.1265/jjh.45.1029 2051628

